# *Terminalia ferdinandiana* Exell. extracts reduce pro-inflammatory cytokine and PGE_2_ secretion, decrease COX-2 expression and down-regulate cytosolic NF-κB levels

**DOI:** 10.1007/s10787-024-01462-7

**Published:** 2024-04-06

**Authors:** Ian E. Cock

**Affiliations:** 1 Centre for Planetary Health and Food Security, Griffith University, Nathan Campus, 170 Kessels Rd, Nathan, QLD 4111 Australia; 2 School of Environment and Science, Griffith University, Nathan Campus, 170 Kessels Rd, Nathan, QLD 4111 Australia

**Keywords:** *Combretaceae*, Kakadu plum, Immuno-modulation, Interleukins, Pro-inflammatory cytokines, Anti-inflammatory activity, COX-2, Prostaglandin E_2_

## Abstract

Based on their high antioxidant capacity and noteworthy phytochemistry, *Terminalia ferdinandiana* fruit and leaves have attracted considerable recent interest for their therapeutic potential. Whilst those studies have reported a variety of therapeutic properties for the fruit, the anti-inflammatory potential of *T. ferdinandiana* has been largely neglected and the leaves have been almost completely ignored. This study investigated the immune-modulatory and anti-inflammatory properties of *T. ferdinandiana* fruit and leaf extracts by evaluating their inhibition of multiple pro- and anti-inflammatory cytokines and chemokines secretion in lipopolysaccharide (LPS)-stimulated and unstimulated RAW 264.7 macrophages using multiplex bead immunoassays and ELISA assays. The methanolic extracts were particularly good immune-modulators, significantly inhibiting the secretion of all the cytokines and chemokines tested. Indeed, the methanolic extracts completely inhibited IL-10, IFN-γ, IL-1β, IL-6, MCP-1, and MIP-2a secretion, and almost completely inhibited the secretion of TNF-α. In addition, the methanolic *T. ferdinandiana* extracts also significantly inhibited cytosolic COX-2 levels (by 87–95%) and the synthesis of the PGE_2_ (by ~ 98%). In contrast, the methanolic extracts stimulated LTB_4_ secretion by ~ 60–90%, whilst the aqueous extracts significantly inhibited LTB_4_ secretion (by ~ 27% each). Exposure of RAW 264.7 cells to the methanolic *T. ferdinandiana* extracts also significantly down-regulated the cytosolic levels of NF-κB by 33–44%, indicating that the immune-modulatory and anti-inflammatory properties of the extracts may be regulated via a decrease in NF-κB transcription pathways. Taken together, these results demonstrate potent anti-inflammatory properties for the extracts and provide insights into their anti-inflammatory mechanisms.

## Introduction

Inflammation is a complex biological response by which the immune system recognises tissue damage and removes harmful stimuli to allow the body to heal. Inflammatory processes may be initiated in response to pathogen infections (bacterial, viral, fungal), localised tissue injuries (e.g. burns, scrapes, insect stings, and wounds), or by exposure to chemicals or radiation. In response to inflammatory stimuli, cells release a myriad of chemical effectors including cytokines, chemokines, prostaglandins and leukotrienes (Libby 2007). Pro-inflammatory cytokines including interleukin (IL)-1β, IL-2, IL-6, as well as interferon gamma (IFN-γ) and tumour necrosis factor alpha (TNF-α), initiate and amplify the early-phase inflammatory events (Kany et al. 2019). Anti-inflammatory cytokines such as IL-10 limit the effects of the pro-inflammatory agents and modulate the inflammatory processes (Meng et al. 2019). Regardless of the trigger stimulus, inflammation induces vasodilation, increases blood flow to the affected area, stimulates the release of further soluble factors that amplify the response, and increases blood vessel permeability, resulting in tissue swelling (Gusev and Zhuravleva 2022). Cells at the site of stimulus release chemotactic agents including IL-8, monocyte chemotactic protein (MCP)-1 and macrophage inflammatory (MIP)-2a, resulting in leukocyte migration to the site of injury (Wong et al. 2010). Specific secreted inflammatory mediators may also activate phospholipase A_2_, which then catalyses arachidonic acid (AA) release from cell membrane lipids (Boi et al. 2022). AA is subsequently metabolised by the cyclooxygenase (COX) and lipoxygenase (LOX) systems to produce further inflammatory mediators, including prostaglandins and leukotrienes (Calder 2020).

Inflammation is a normal part of the body’s response to injury and is generally beneficial, although severe and/or prolonged inflammation may cause pain and discomfort and pharmaceutical intervention may be required to alleviate these effects. Non-steroidal anti-inflammatory drugs (NSAIDs) are the most frequently used class of drugs used to treat inflammation. Steroidal anti-inflammatory drugs (SAIDs) are also effective and may be used for short periods to treat particularly severe inflammation. However, the NSAIDs and SAIDs are both toxic and induce multiple side effects including heartburn, nausea, hypertension (and, therefore, increased risks of strokes and coronary disease), headache, liver and kidney toxicity, and gastric ulcers (Brune and Patrignani 2015; Roubille et al. 2015). Safe and effective new therapies to treat inflammation are needed and further research to develop new therapies is required. Medicinal plants and the phytochemicals isolated from them provide useful targets for the anti-inflammatory therapy development. Indeed, several recent studies have targeted traditional herbal medicines as anti-inflammatory therapeutics and have reported noteworthy anti-inflammatory properties (Ryan et al. 2021; Anandakumar et al. 2014; de Cássia da Silveira e Sá et al. 2013).

*Terminalia ferdinandiana* Exell. (Family Combretaceae; commonly known as Kakadu plum, gubinge, murunga, billygoat plum) is a medium-sized deciduous tree that is endemic to the northern and north-western tropical woodland regions of Australia. The fruit of this species has an extremely high antioxidant capacity, with ascorbic acid contents reported up to 6% of the wet weight (Shalom and Cock 2018; Konczak et al. 2010). In addition, *T. ferdinandiana* fruit (Rayan et al. 2015; Sirdaarta et al. 2015) and leaves (Shalom and Cock 2018) are rich in tannins, which have been linked with many of the therapeutic uses of this species (Cock 2015). Due to its high antioxidant activity and its noteworthy phytochemistry, *Terminalia ferdinandiana* fruit have attracted considerable recent interest and the anticancer (Shalom and Cock 2018; Tan et al. 2011a, b), antibacterial (Cheesman et al. 2019; Wright et al. 2019; McManus et al. 2017) and anti-giardial (Cock and Rayan 2020; Rayan et al. 2015) activities have been reported. Despite these reports, the anti-inflammatory properties are yet to be thoroughly evaluated. Some studies reported that fractions prepared from *T. ferdinandiana* fruit extracts significantly up-regulated the Nrf2/Keap1 ratio in Hep G2 cells and inhibited the expression of COX-2 in LPS activated macrophages (Tan et al. 2011a, b). The authors postulated that these effects were mediated via NF-κB modulation. Notably, those studies only evaluated the anti-inflammatory effects of the fruit and the effects of the leaves were not evaluated. This study extends the earlier reports by evaluating the immune-modulatory and anti-inflammatory activities of *T. ferdinandiana* fruit and leaf extracts by quantifying their modulation of anti-inflammatory and pro-inflammatory cytokines and chemokines secretion in a LPS-stimulated and unstimulated murine RAW 264.7 macrophages. In addition, the effects of the extracts on COX-2 expression and the secretion of PGE_2_ were examined to confirm the results of the earlier study. Modulation of LTB_4_ secretion is reported herein for the first time. The cytosolic levels of NF-κB were also examined to further determine how the *T. ferdinandiana* extracts modulate the levels of this inflammatory regulator.

## Materials and methods

### Plant material and extraction

*Terminalia ferdinandiana* Exell. plant materials were provided by Wild Harvest, Northern Territory (Australia). Fresh fruit was pulped within 24 h of harvest and the pulp was frozen and stored at −10 °C for transport and until it was processed. Mature green leaves were harvested and immediately transported for processing. The *T. ferdinandiana* plant materials were separately dehydrated using a food dehydrator (Sunbeam) at 23 °C. The desiccated plant materials were subsequently stored at −30 °C. Voucher specimens (dried fruit pulp: KP2014GD; leaves: KP2015LA) are stored at Griffith University in the School of Natural Sciences. The dried plant materials were freshly ground into coarse powders prior to use. Individual 1 g masses of ground fruit and leaf powders was extensively extracted in 50 mL of either AR grade methanol (Ajax Fine Chemicals, Australia) or sterile deionised water for 24 h at 4 °C. Particulate matter was removed by filtering the extracts using Whatman No. 54 filter paper. The methanolic extracts were subsequently air dried in the shade at room temperature, whilst the aqueous extracts were dried in a vacuum oven at 50 °C. The resultant dried extracts were dissolved in 10 mL aqueous 0.5% DMSO and sterilised by filtration through a 0.22 μm filter (Sarstedt). The extracts were stored at 4 °C until use.

### Positive control for anti-inflammatory assays

Powdered turmeric capsules (Super Strength Bio Turmeric 3100) were obtained from Healthy Care, Australia (Batch no. 750710). Individual capsules contained 155 mg of dried turmeric that was standardised by the manufacturer to contain the equivalent to 100 mg curcumin. The turmeric powder contents was removed from a single capsule and weighed to confirm the mass. The powder was dissolved in 4 mL DMSO and 28 mL of sterile distilled water was added to prepare a 5 mg/mL (12.5% DMSO) stock turmeric solution. The turmeric stock was freshly diluted immediately before use in the bioassays and used as a positive control at 1.25 mg/mL (containing 3.12% DMSO).

### Cell culture and treatment

Murine macrophages (RAW 264.7: TIB-71) and human dermal fibroblasts (HDF; PCS-201-010) were obtained from the American Type Culture Collection (ATCC). Both cell lines were cultured in Dulbecco’s Modified Eagle’s medium (DMEM, Gibco), supplemented with 10% fetal bovine serum (FBS, Gibco) and 100 µg/mL penicillin/streptomycin (Gibco). The cells were maintained at 37 °C and 70% humidity in a 5% CO_2_ incubator until approximately 80% confluence was achieved (evaluated microscopically). Both cell lines were passaged twice a week, and four passages were conducted before experiments were commenced.

### Measurement of cell viability by MTS assay

MTS (3-(4,5-dimethylthiazol-2-yl)-5-(3-carboxymethoxyphenyl)-2-(4-sulfophenyl)-2H-tetrazolium) assays were performed using standard methods (Shalom and Cock 2018) to evaluate cell viability following exposure to the extracts. These assays were used for evaluating the safety of the extracts for therapeutic use, and to determine appropriate extract doses with low toxicity for biological screening. The assay was performed following standard protocols, with the following adaptions. A 100 µL volume of cells (containing approximately 1 × 10^6^ cells) was added into each well of sterile 96-well microtitre plates and 100 μL of test extract, turmeric suspension, 10% DMSO-positive control, or fresh DMEM media (untreated control) were added in triplicate, and three independent experiments were performed (*n* = 9). The plates were incubated for 24 h at 37 °C in a humidified 5% CO_2_ environment. After the 24 h incubation, the cells were exposed to 100 µL of doubling serial dilutions of the plant extracts or controls, and then the plates were incubated for another 24 h at 37 °C. Fresh DMEM media (containing 3% DMSO) was added to the cells instead of extract, as a negative control, which resulted in a 1.5% DMSO concentration in the assay. Cells exposed to 10% DMSO served as positive controls for the assay. Following a further 48 h incubation, the spent media (containing the extracts or controls) was aspirated and discarded. Then, 100 µL of fresh DMEM media and 25 µL of CellTiter 96^®^ Aqueous One Solution Reagent (Promega, Australia) were added. The cells were then incubated for another 1.5 h under the same conditions. The MTS solution colour change was observed at 490 nm using a Molecular Devices Spectra Max M3 plate reader as a measure of cellular viability.

### Cytokine multiplex bead assay

RAW 264.7 murine macrophages were cultured in fresh DMEM media supplemented with 10% heat-inactivated fetal bovine serum (Gibco, Thermo Fisher Scientific, MA, USA) and 100 U/mL penicillin/100 μg/mL streptomycin (Invitrogen). The cells were incubated in a 5% CO_2_ atmosphere at 37 °C. The immune-modulatory effects of the *T. ferdinandiana* extracts (2.5 mg/mL) and the turmeric control (1.25 mg/mL) were evaluated in both unstimulated RAW 264.7 macrophages (as a measure of basal cytokine release following extract exposure), and in lipopolysaccharide (LPS; 100 ng/mL; Sigma-Aldrich, Australia) stimulated cells. DMED media (100 μL containing approximately 1 × 10^6^ cells) were used to seed all wells of 6-well flat-bottom culture plates. The cells were pre-treated with the tests (2.5 mg/mL of the *T. ferdinandiana* extracts) or of the turmeric control (1.25 mg/mL) and incubated for 3 h. LPS (for the LPS-stimulated cells) or fresh DMEM media (for the unstimulated tests) was then added and the plates were incubated for a further 48 h at 37 °C in a 5% CO_2_ incubator at 70% humidity. The culture supernatants were then collected and used for cytokine analysis. Aliquots were also retained and stored at −80 °C for quantification of PGE_2_ and LTB_4_ secretion.

Customised Microplex Bead assays kits coated with antibodies against murine IL-2, IL-10, IFNγ, IL-1β, IL-6, MCP-1, MIP-2α and TNFα were purchased from ThermoFisher, Australia. The multiplex bead assays were performed and the levels of secreted cytokines and chemokines were quantified as per the manufacturers’ instructions at Cardinal Bioresearch, Australia using a Luminex 200 flow-cytometry system (Milliplex^®^, Australia). Sample fluorescence intensity (MFI) was used to quantify the cytokine concentrations (in pg/mL) by comparison to cytokine standard curves. The cytokine secretions levels are expressed as a % of the untreated control RAW cells. All tests were performed in two independent experiments (*n* = 2) and are expressed as mean ± standard deviation.

### Preparation of cell lysates for COX-2 and NF-κB ELISA analysis

Murine RAW 264.7 macrophages were cultured in fresh DMEM media and the adherent cells were dislodged and washed using ice-cold phosphate-buffered saline solution (PBS). The cells were lysed in 10 mM Tris pH 7.4 (Invitrogen), 150 mM HCl, 1 mM EDTA, 0.5% SDS, 0.04 mM NAF, and 1% TritonX-100, containing protease inhibitor cocktail (Merck) to obtain the total cellular protein content. The cell lysates were centrifuged at 20,000 *g* and 4 °C for 40 min to remove cellular debris. The concentration of protein in the supernatant was determined using a Pierce™ BCA Protein Assay Kit (Thermo Fisher Scientific, Australia) following the manufacturer’s instructions.

### Cyclooxygenase-2 (COX-2) assay

The effects of the extracts and the turmeric control on COX-2 levels in the murine RAW 264.7 macrophages were evaluated using a murine COX-2 ELISA Kit (ab210574, Abcam, Australia) following the manufacturer’s instructions. The protein concentration of all supernatants was adjusted to 50 μg/mL for all tests. Test samples and controls were individually aspirated into individual wells of the 96-well plate provided in the assay kit, which were coated with the antibodies specific for the murine COX-2 enzyme. The assays were performed following the manufacturer’s protocol, and the absorbance was measured in a Molecular Devices Spectra Max M3 plate reader at 450 nm. The results were calculated in ng/mL and are presented herein as the mean percentage of the untreated control ± standard deviation of triplicates for each treatment (*n* = 3).

### Nuclear factor kappa-B (NF-κB) assay

The effects of the extracts and turmeric control on NF-κB were measured using murine NF-κB p65 (pS536) ELISA kits (ab176647, Abcam, Australia) as per the manufacturer’s instructions. The protein concentrations of the test and control cell lysates were diluted to 500 μg/mL each, and were added to separate wells on the 96-well plates (coated with antibodies specific to the p65 region of murine NF-κB). Absorbances were measured at 450 nm in a Molecular Devices Spectra Max M3 plate reader. All test and control samples were repeated in triplicate (*n* = 3) and the results are expressed as a percentage of the untreated controls ± standard deviation.

### PGE_2_ and LTB_4_ assays

Cell culture supernatants (prepared as described in “Cytokine multiplex bead assay”) were used to quantify PGE_2_ and LTB_4_ levels using pre-prepared ELISA kits (ab287802, Abcam, Australia for PGE_2_ and EHLTB4, Thermo Fisher Scientific, Australia for LTB_4_, respectively). All tests were performed following manufacturer’s instructions. The absorbance at 450 nm was measured using a Molecular Devices Spectra Max M3 plate reader, and used to calculate the concentrations in pg/mL. All test and control samples were repeated in triplicate (*n* = 3) and are presented as the percentage of the untreated control values ± standard deviation.

### LC–MS fingerprint analysis

Chromatographic separations and compound identifications were performed using protocols developed in our group (Shalom and Cock 2018). Briefly, 2 µL of each extract was injected individually onto an Agilent 1290 HPLC system equipped with a Zorbax Eclipse plus C18 column (2.1 × 100 mm, 1.8 µm particle size) and eluted at 0.7 mL/min. Extract components were separated using a gradient, which consisted of a 5 min isocratic phase with 95% ultrapure water, followed by a 25 min gradient to 95% acetonitrile gradient. Mass spectrometry analysis was performed using an Agilent 6530 QTOF (quadrupole time-of-flight) spectrometer coupled to a Jetstream electrospray ionisation source in negative ionisation mode. The individual compounds detected were screened against two accurate mass databases to putatively identify the compounds: the Metlin metabolomics database (24,768 compounds), and a database of plant compounds generated for this study (800 compounds).

## Results

### Cytotoxicity profiles against RAW 264.7 murine macrophages and human fibroblast (HDF) cell lines

**Fig. 1:**
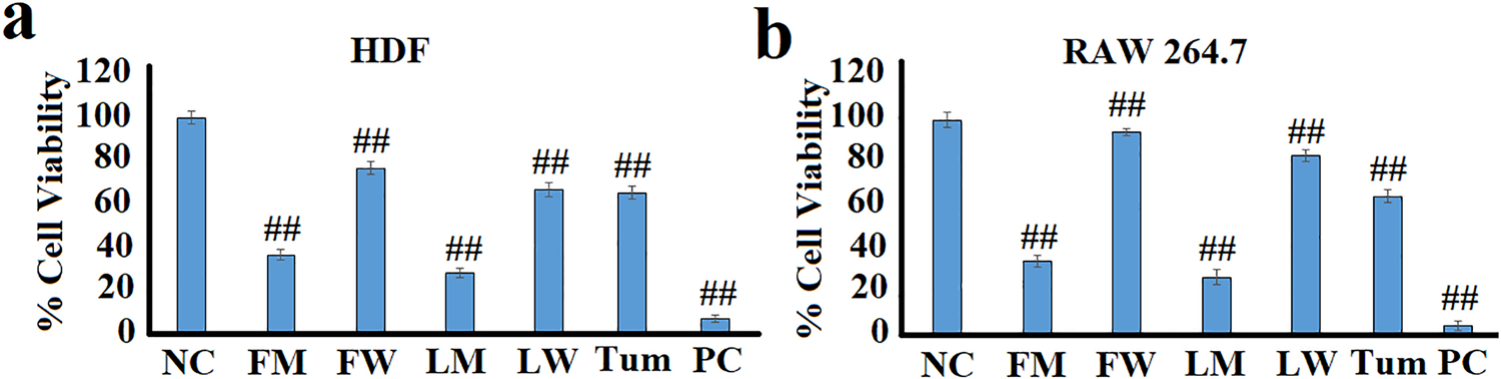
The cytotoxic effects of *T. ferdinandiana* fruit and leaf extracts and a turmeric control on **a** human dermal fibroblasts and **b** RAW 264.7 cell lines at 5 mg/mL as determined by MTS cell viability assays. *NC* negative control (media); FM = methanolic fruit extract; FW = aqueous fruit extract; LM = methanolic leaf extract; LW = aqueous leaf extract; Tum = turmeric control (1.25 mg/mL); PC = 10% DMSO-positive control. Data are represented as mean values of triplicate results ± standard deviation. ## represents results that are significantly different to the negative control (*p* < 0.005)

The cytotoxicity of the *T. ferdinandiana* fruit and leaf extracts was initially screened at 5 mg/mL against human dermal fibroblasts (HDF; Fig. 1a) to evaluate their suitability for therapeutic use as fibroblasts are one of the most widely used cell lines for cytotoxicity evaluations (McGaw et al. 2014). The extracts were also screened against murine RAW 264.7 macrophage to determine the concentrations to be used for testing in the cytokine, COX-2, PGE_2_, LTB_4_ and NF-κB assays (Fig. 1b). Macrophages produce inflammatory mediators during an immune response (Fullerton and Gilroy 2016). Therefore, HDF and RAW 264.7 cells were considered relevant in vitro models for the toxicity evaluations in this study.

The aqueous extracts were substantially less cytotoxic towards both cell lines, compared to the corresponding methanolic extracts. Indeed, exposure to the aqueous fruit and leaf extracts (5 mg/mL) decreased HDF cell viability by approximately 23% and 33%, respectively, compared to the untreated (media) control (Fig. 1a). The RAW 264.7 cells were even less affected by the aqueous fruit and leaf extracts, with decreases in cell viability of approximately 5% and 16%, respectively. Whilst these decreases in cell viability are statistically significant, the viability for all cells exposed to the aqueous extracts were substantially greater than 50%. Therefore, the aqueous *T. ferdinandiana* fruit and leaf extracts were considered nontoxic at 5 mg/mL and were not tested at further dilutions. In contrast, both the fruit and leaf methanolic extracts induced substantially greater decreases in cell viability in both cell lines. Indeed, exposure to the methanolic fruit and leaf extracts resulted in HDF cell viabilities of approximately 36% and 28%, respectively (Fig. 1a). Similar decreases in RAW 264.7 viability were also noted for the fruit and leaf methanolic extracts (~ 34% and 27% of the untreated control cell viability, respectively). The turmeric control decreased cell viability in the HDF and RAW 264.7 cells by ~ 36% and 38%, respectively.

Due to the greater decrease in cell viability noted for the methanolic extracts, their toxicity was further evaluated by screening a dilution series for each extract and calculating LC_50_ values (Table 1). Plant extracts with LC_50_ values < 1 mg/mL were considered to be toxic in this study. Notably, the LC_50_ values were ≥ 2.5 mg/mL for all extracts. Therefore, all the *T. ferdinandiana* extracts were deemed to be nontoxic towards HDF and RAW 264.7 cells and 2.5 mg/mL was selected as the test extract concentration for the cytokine, COX-2, PGE_2_, LTB_4_ and NF-κB inhibition assays.

**Table 1:** The LC_50_ values (μg/mL) of *T. ferdinandiana* extracts against RAW 264.7 murine macrophage and human dermal fibroblasts (HDF)

Plant part	Extract	LC_50_ (μg/mL)	
		HDF	RAW
Fuit	Methanol	3333	3333
	Water	NA	NA
Leaf	Methanol	2500	3333
	Water	NA	NA

NA indicates extracts for which the LC_50_ values were not calculated as no toxicities were observed in the cell viability assay

### Modulation of cytokine secretion in RAW 264.7 cells by *T. ferdinandiana* extracts

The extracts were screened for their effects on cytokine secretion in both unstimulated and LPS-stimulated RAW 264.7 cells to evaluate their immune-modulatory effects. The cytokines screened in our study were selected to test the effects on both anti-inflammatory (IL-10) and pro-inflammatory cytokines (IFNγ, IL-1β, IL-6, and TNF-α) (Kany et al. 2019). IL-2 was included as it has both anti- and pro-inflammatory effects under different conditions (Kolios et al. 2021; Sharma et al. 2011). In addition, the chemokines MCP-1 and MIP-2a were included to evaluate the potential of the extracts to regulate the recruitment of monocytes to the site of the inflammation (Wong et al. 2010).

### Effect of the extracts on cytokine secretion in unstimulated RAW 264.7 cells

**Fig. 2:**
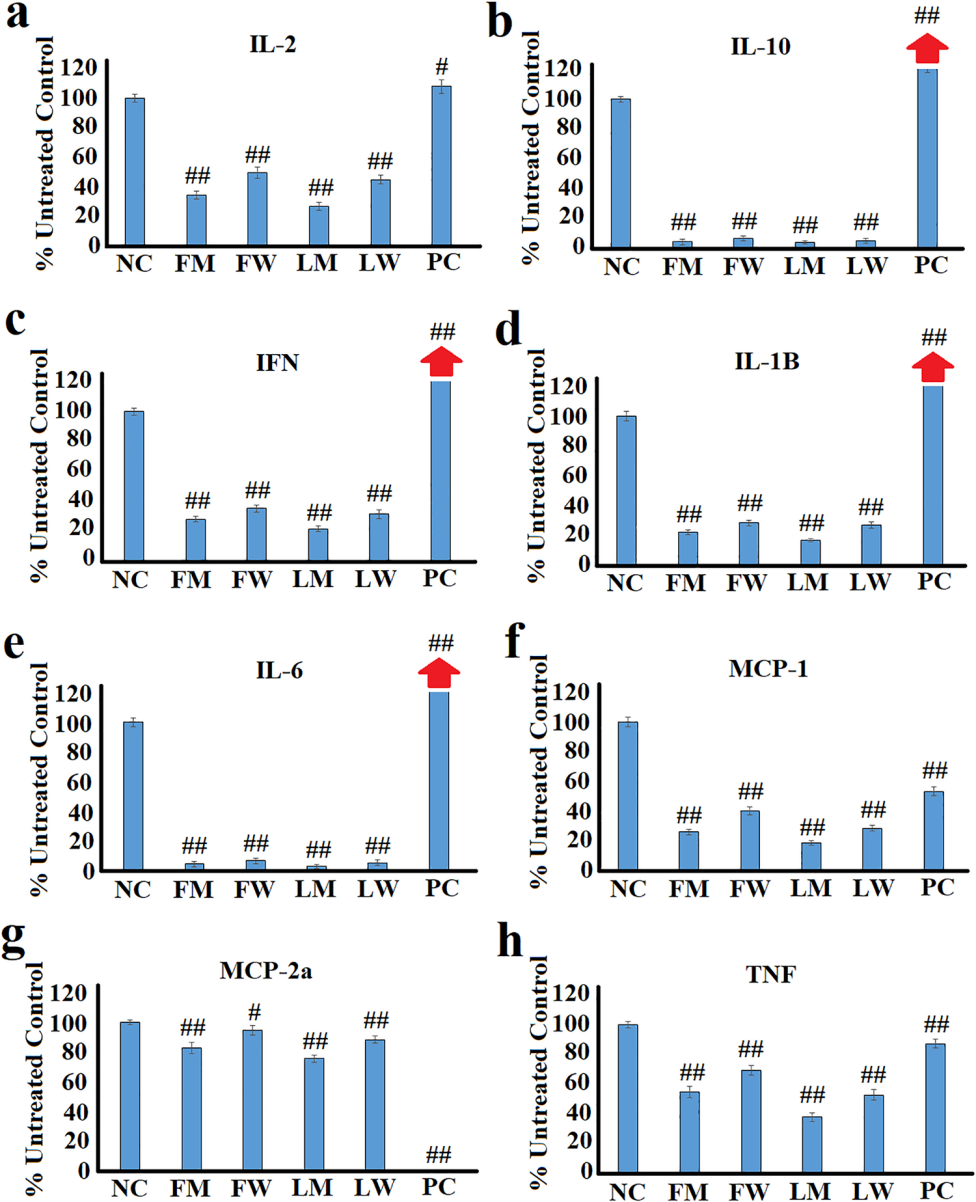
The effects of the *T. ferdinandiana* fruit and leaf extracts (2.5 mg/mL) on secretion of **a** IL-2; **b** IL-10; **c** IFNγ; **d** IL-1β; **e** IL-6; **f** MCP-1; **g** MIP-2α; and **h** TNFα in unstimulated RAW 264.7 cells. Arrows indicate plant extracts that induce cytokine levels substantially greater than 120%. NC = untreated negative control (media); FM = methanolic fruit extract; FW = aqueous fruit extract; LM = methanolic leaf extract; LW = aqueous leaf extract; PC = turmeric positive control (1.25 mg/mL). Data are represented as mean values of duplicate results ± standard deviation. # and ## represent results that are significantly different to the negative control at *p* < 0.01 and *p* < 0.005, respectively

To investigate whether the *T. ferdinandiana* fruit and leaf extracts modulate inflammatory mediators in the absence of a pro-inflammatory stimulus, RAW 264.7 macrophages were treated with the extracts, without prior LPS stimulus (Fig. 2). Interestingly, treatment with the extracts decreased the secretion of both pro-inflammatory and anti-inflammatory cytokines, indicating that they may be affecting a general regulatory mechanism. IL-2 (Fig. 1a) and IL-10 (Fig. 1b) were included in this study as they both have anti-inflammatory effects (IL-2 also has pro-inflammatory properties) (Kolios et al. 2021; Sharma et al. 2011). Therefore, increased secretion of these cytokines is associated with anti-inflammatory activity. Interestingly, all extracts significantly inhibited the secretion of these cytokines. The extracts were potent inhibitors of IL-10, with secretion inhibited by > 95% by all extracts. Similarly, IL-2 was also inhibited, albeit to a lesser extent (50–73% inhibition compared to the untreated control). Whilst this may not result in pro-inflammatory effects, the decreased levels of anti-inflammatory cytokines cells may confer a decreased ability for the cells to respond to inflammation. In contrast, the turmeric control induces substantial increases in IL-10 secretion (to ~ 122% of the untreated control), indicating that turmeric treatment increases the cells ability to respond to inflammation.

The *T. ferdinandiana* extracts also significantly (*p* < 0.005) down-regulated the secretion of the pro-inflammatory cytokines IFN-γ (Fig. 1c), IL-1β (Fig. 1d), IL-6 (Fig. 1e) and TNF-α (Fig. 1h), demonstrating that the *T. ferdinandiana* extracts have immune-modulatory and anti-inflammatory effects. The extracts were particularly good inhibitors of IL-6 secretion (Fig. 1e), with between 93 and 97% inhibition noted, in comparison to the untreated control. The extracts also significantly inhibited the secretion of all other pro-inflammatory cytokines (generally inhibited by 65–83% of the untreated control levels). This indicates that these extracts have anti-inflammatory effects. Notably, the turmeric control had the opposite effect to that of the *T. ferdinandiana* extracts. Indeed, turmeric stimulated pro-inflammatory cytokine secretion in RAW 264.7 macrophages by up to 163% of the untreated control value (for IFN-γ). Exposure to the *T. ferdinandiana* extracts also reduced the secretion of the MCP-1 (Fig. 1f) and MIP-2a (Fig. 1g) chemokines. Notably the turmeric control was a potent inhibitor of chemokine secretion, with a complete inhibition of MIP-2a noted.

### Effect of the extracts on cytokine secretion in LPS-stimulated RAW 264.7 cells

**Fig. 3:**
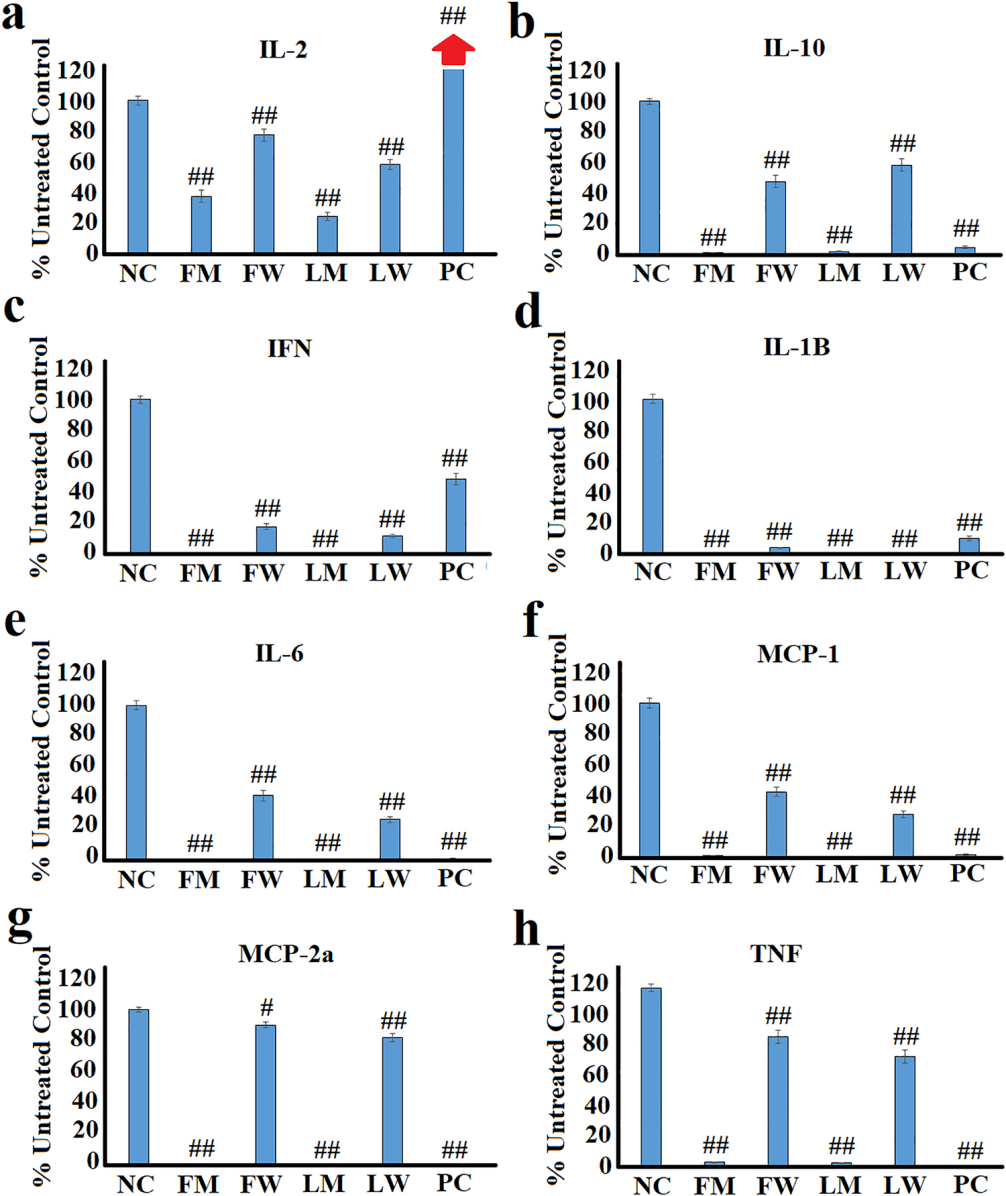
The effects of the *T. ferdinandiana* fruit and leaf extracts (2.5 mg/mL) on secretion of **a** IL-2; **b** IL-10; **c** IFNγ; **d** IL-1β; **e** IL-6; **f** MCP-1; **g** MIP-2α; and **h** TNFα in LPS-stimulated (100 ng/mL) RAW 264.7 cells. Arrows indicate percentage inhibition of plant extracts that is substantially greater than 120% scale of the graphs. NC = untreated negative control (media); FM = methanolic fruit extract; FW = aqueous fruit extract; LM = methanolic leaf extract; LW = aqueous leaf extract; PC = turmeric positive control (1.25 mg/mL). Data are represented as mean values of duplicate results ± standard deviation. # and ## represent results that are significantly different to the negative control at *p* < 0.01 and *p* < 0.005, respectively

The *T. ferdinandiana* extracts were also tested against LPS-stimulated RAW 264.7 macrophages to evaluate their ability to modulate immune-modulatory markers during inflammation (Fig. 3). Notably, the *T. ferdinandiana* extracts were potent inhibitors of the secretion of all anti-inflammatory and pro-inflammatory cytokines, as well as the chemokines. The methanolic extracts were substantially more potent inhibitors of cytokine secretion than the aqueous extracts were. Interestingly, the methanolic fruit and leaf extracts blocked 41–52% of IL-2 (Fig. 1a) and 96–98% of the secretion of IL-10 (Fig. 2b), respectively, from the RAW 264.7 cells, indicating they may potentiate the inflammatory effects of LPS. The turmeric control also significantly inhibited IL-10 secretion. In contrast, turmeric stimulated IL-2 secretion compared to the untreated LPS-stimulated control. The aqueous extracts also significantly inhibited the secretion of IL-2 and IL-10, although the inhibition was substantially less potent.

The *T. ferdinandiana* fruit and leaf extracts were substantially more potent inhibitors of secretion of the pro-inflammatory cytokines and the chemokines from RAW 264.7 cells. Indeed, the methanolic fruit and leaf extracts completely inhibited the secretion of IFN-γ (Fig. 2c), IL-1β (Fig. 2d), IL-6 (Fig. 2e), as well as inhibiting TNF-α (Fig. 2h) secretion by ~ 98%. Notably, the inhibition of pro-inflammatory cytokine secretion by the methanolic extracts was similar to that determined for the turmeric control. The aqueous extracts also significantly inhibited pro-inflammatory cytokine secretion (*p* < 0.005), albeit by a substantially lower percentage than noted for the methanolic extracts. The potent inhibition of the pro-inflammatory cytokines indicates that the *T. ferdinandiana* extracts have anti-inflammatory effects. Similarly, the *T. ferdinandiana* extracts (and the turmeric control) completely blocked the secretion of the MCP-1 (Fig. 2f) and MIP-2a chemokines (Fig. 2g) from RAW 264.7 macrophages.

### The effect of *T. ferdinandiana* extracts on COX-2 levels in RAW 264.7 cells

**Fig. 4:**
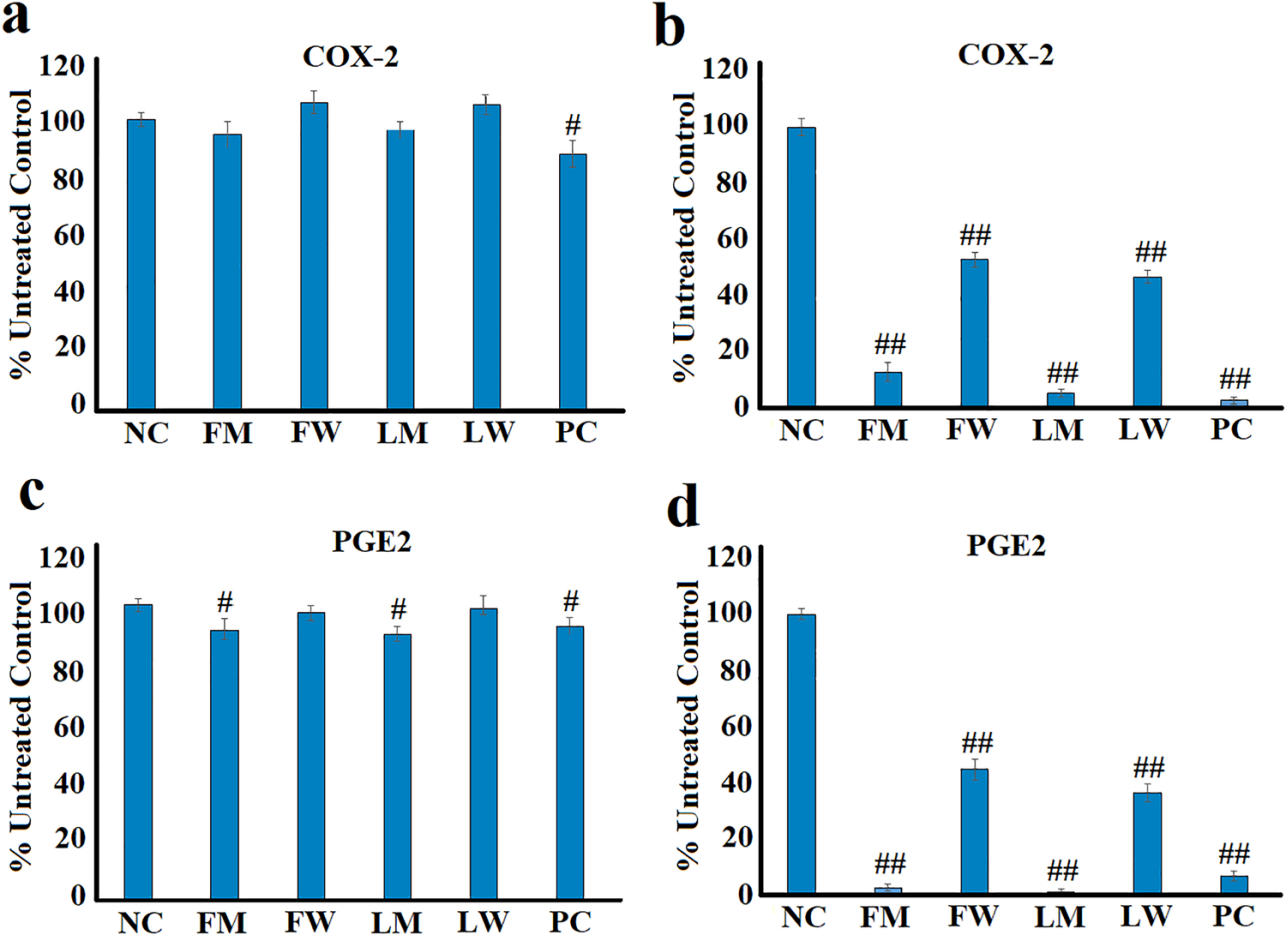
The effects of the *T. ferdinandiana* fruit and leaf extracts on COX-2 levels in **a** unstimulated and **b** LPS-stimulated (100 ng/mL) RAW 264.7 cells, as well as PGE_2_ levels in **c** unstimulated and **d** LPS-stimulated (100 ng/mL) RAW 264.7 cells. *NC* negative control (media); FM = methanolic fruit extract; FW = aqueous fruit extract; LM = methanolic leaf extract; LW = aqueous leaf extract; PC = turmeric positive control (1.25 mg/mL). Data are represented as mean values of triplicate results ± standard deviation. # and ## represent results that are significantly different to the negative control at *p* < 0.01 and *p* < 0.005, respectively

The cytosolic levels of COX-2 in the unstimulated RAW 264.7 macrophages were unaffected by treatment with the *T. ferdinandiana* leaf and fruit extracts when tested at a concentration of 2.5 mg/mL (Fig. 4a). Indeed, no significant differences were noted between the cells exposed to the *T. ferdinandiana* extracts and the untreated control cells. This is perhaps not surprising as the COX-2 enzyme is inducible and its synthesis is up-regulated during inflammation, whilst its levels are generally low in unstimulated cells (Ju et al. 2022; Mohsin and Irfan 2019). In contrast, the turmeric positive control significantly down-regulated the cytosolic COX-2 level in the RAW 264.7 macrophages, although the COX-2 levels were only decreased by approximately 9% (*p* < 0.01).

In contrast, all the *T. ferdinandiana* extracts substantially reduced COX-2 levels in LPS-stimulated RAW 264.7 macrophages (Fig. 4b). The methanolic fruit and leaf extracts were particularly good at reducing COX-2 levels, decreasing cytosolic COX-2 levels by approximately 87% and 95% compared to the untreated LPS-stimulated control, respectively. The aqueous extracts also significantly reduced COX-2 levels in RAW 264.7 cells (*p* < 0.005), with reductions of approximately 47% and 43%, respectively, compared to the untreated LPS-stimulated control. The turmeric positive control had profound effects on COX-2 levels, indicating that the assay was functioning correctly. Indeed, turmeric treatment resulted in a 98% reduction in COX-2 levels compared to the untreated control.

### The effect of ***T. ferdinandiana*** extracts on PGE_2_ secretion in RAW 264.7 cells

Prostaglandin E_2_ is produced from arachidonic acid in a series of reactions, starting with an oxygenation reaction catalysed by the COX enzymes (Park et al. 2006). Therefore, we quantified the levels of PGE_2_ secreted by both the unstimulated and the LPS-stimulated RAW 264.7 macrophages. As noted for COX-2, the *T. ferdinandiana* fruit and leaf extracts had minimal effects on PGE_2_ secretion in non-LPS-stimulated RAW 264.7 macrophages (Fig. 4c). Indeed, neither the aqueous fruit nor the leaf extracts significantly affected the secretion of PGE_2_ in the unstimulated cells. Whilst significant reductions (*p* < 0.01) in PGE_2_ secretion were noted in response to treatment with the methanolic extracts, the levels were only reduced by approximately 8% compared to the untreated control. Similar decreases in PGE_2_ secretion were also noted following turmeric treatment.

In contrast, the *T. ferdinandiana* extracts had profound effects on the secretion of PGE_2_ in LPS-stimulated cells (Fig. 4d). The methanolic fruit and leaf extracts were particularly effective, inhibiting PGE_2_ secretion by approximately 97% and 99%, respectively, compared to the untreated LPS-stimulated control. The inhibition by the methanolic extracts compared favourably to the inhibition noted for the turmeric positive control (~ 93%). The aqueous fruit (~ 55% inhibition) and leaf extracts (~ 65% inhibition) were also good inhibitors of PGE_2_ secretion, although substantially less potent than the methanolic extracts (based on the % inhibition compared to the untreated LPS-stimulated control).

### The effect of ***T. ferdinandiana*** extracts on LTB_4_ secretion in RAW 264.7 cells

LTB_4_, another pro-inflammatory mediator, is secreted by macrophages following inflammatory stimuli (as reviewed in Haeggstrom and Funk 2011). It is a potent chemotactic agent, with activity more than three orders of magnitude higher than histamine. LTB_4_ is a mediator of pathogenesis for multiple acute and chronic inflammatory conditions including asthma, atherosclerosis, cancer, dermatitis, inflammatory bowel disease, nephritis, psoriasis and rheumatoid arthritis (He et al. 2020; Liu and Yokomizo 2015). For this reason, the production and secretion of LTB_4_ is a target for the treatment of inflammation. Furthermore, the activity of lipoxygenase (LOX) enzymes (and, therefore, the synthesis of leukotrienes, including LTB_4_) is linked with COX enzyme activity. The inhibition of COX activity makes more arachidonic acid (AA) available for LOX catalysis, which may result in increased secretion of leukotrienes, including LTB_4_ (Calder 2020; Saini et al. 2020). It was, therefore, deemed relevant to assess the effects of the *T. ferdinandiana* extracts on LTB_4_ secretion in RAW 264.7 cells.

**Fig. 5:**
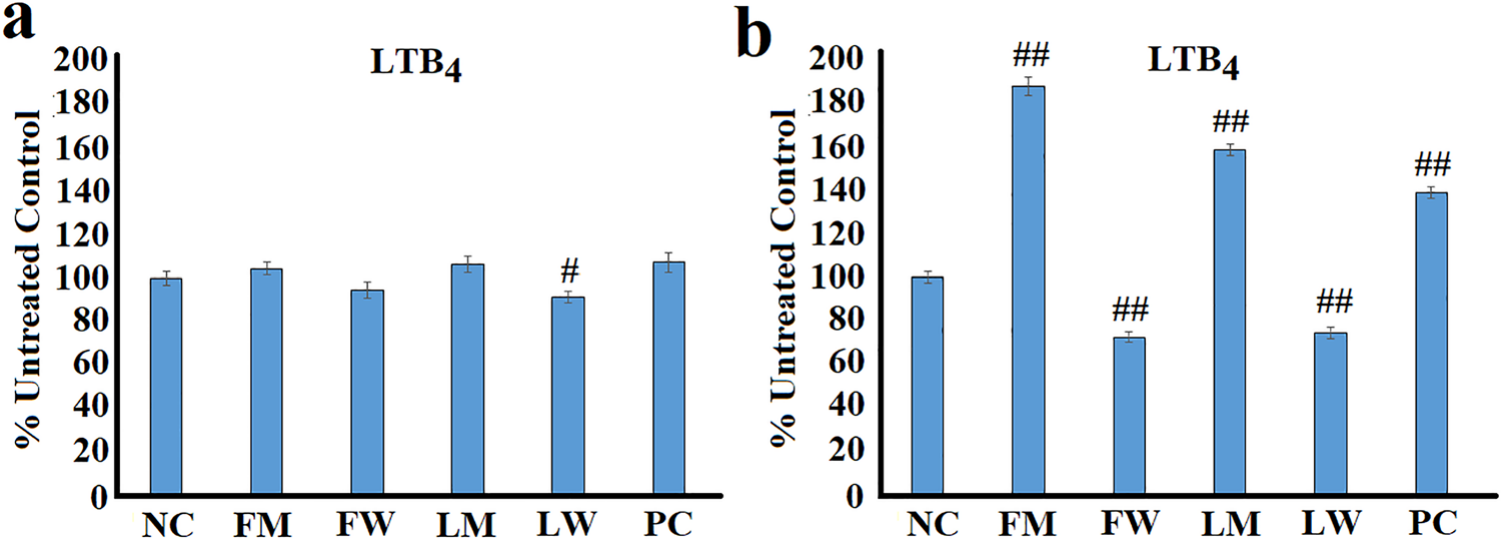
The effects of the *T. ferdinandiana* fruit and leaf extracts on LTB_4_ levels in **a** unstimulated and **b** LPS-stimulated (100 ng/mL) RAW 264.7 cells. *NC* negative control (media); FM = methanolic fruit extract; FW = aqueous fruit extract; LM = methanolic leaf extract; LW = aqueous leaf extract; PC = turmeric positive control (1.25 mg/mL). Data is represented as mean values of triplicate results ± standard deviation. # and ## represent results that are significantly different to the negative control at *p* < 0.01 and *p* < 0.005, respectively

Only the aqueous leaf *T. ferdinandiana* extract had significant effects on LTB_4_ secretion in unstimulated RAW 264.7 macrophages (Fig. 5a). Although the aqueous leaf extract did significantly inhibit LTB_4_ secretion (*p* < 0.01), relatively low inhibitory activity (~ 8%) was noted. Furthermore, the turmeric control also had relatively minor effects on unstimulated RAW 264.7 cells, increasing LTB_4_ secretion by approximately 7%. It is noteworthy that the levels of LTB_4_ secreted in the untreated cells was low and near the detection threshold of this assay (results not shown), and therefore, these differences may not be indicative of inflammo-modulatory activity of the aqueous leaf extract and the turmeric control, and may instead be due to minor fluctuations in the low levels of this lipid. Further work is required to verify whether the trends noted herein demonstrate inflammo-modulation.

The LPS-stimulated RAW 264.7 macrophages secreted substantially higher amounts of LTB_4_ (results not shown), and therefore the effects of the *T. ferdinandiana* extracts were more pronounced (Fig. 5b). An interesting trend was evident: treatment with the methanolic extracts and the turmeric control significantly increased LTB_4_ secretion (*p* < 0.005), indicating pro-inflammatory effects via increased LTB_4_ production. Interestingly, these findings contrast to the effects of the extracts (and turmeric) against cytosolic COX-2 levels (Fig. 4b) and PGE_2_ secretion (Fig. 4d), where substantial decreases were evident. As both COX and LOX enzymes use AA as a substrate, decreased COX-2 synthesis (and the subsequent decreased synthesis of PGE_2_) may make more AA available to be converted to LTB_4_ by LOX, thereby accounting for the increased secretion of LTB_4_. The effects of the aqueous extracts were substantially different, with significant inhibition of LTB_4_ secretion (*p* < 0.005) noted for the aqueous fruit and leaf extracts, with decreases of approximately 27% and 25%, respectively, in comparison with the LPS-stimulated untreated control. Interestingly, the aqueous extracts were substantially less effective inhibitors of COX-2 levels and PGE_2_ secretion in LPS-stimulated RAW 264.7 cells (Fig. 4), and this may decrease the amount of AA available to the cells (compared to the methanolic extracts), thereby reducing the production and secretion of LTB_4_.

### Effect of the extracts on cytosolic NF-κB levels in LPS-stimulated RAW 264.7 cells

**Fig. 6:**
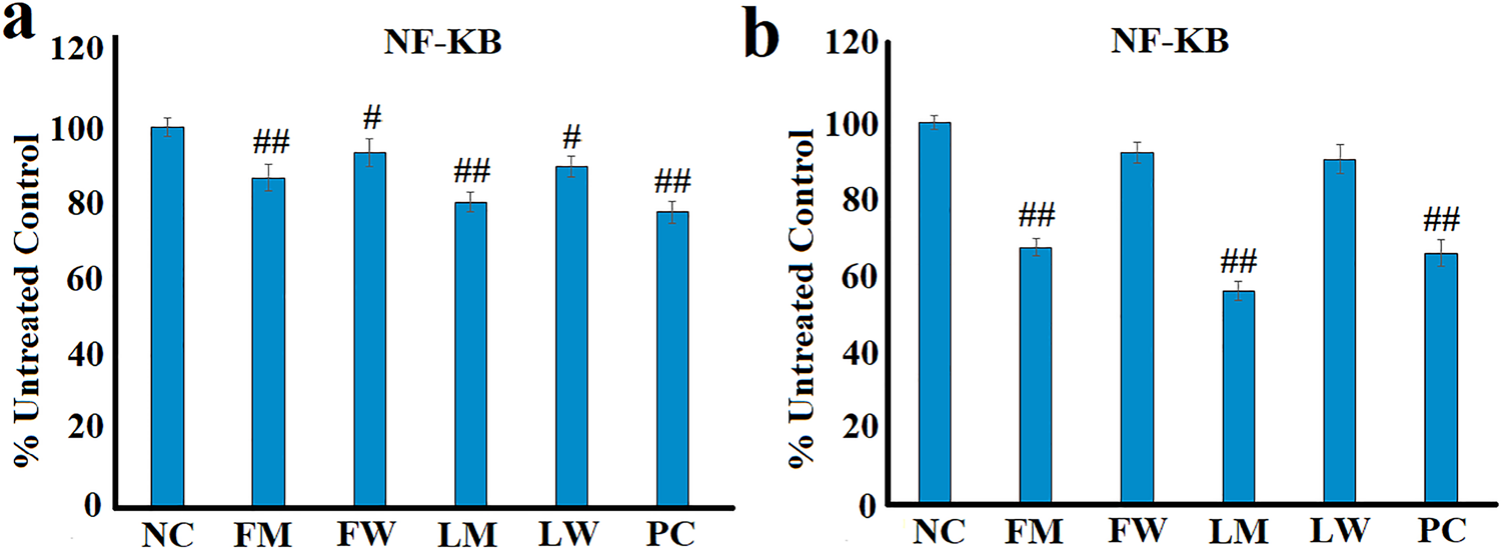
The effects of the *T. ferdinandiana* fruit and leaf extracts on NF-κB levels in **a** unstimulated and **b** LPS-stimulated (100 ng/mL) RAW 264.7 cells. *NC* negative control (media); FM = methanolic fruit extract; FW = aqueous fruit extract; LM = methanolic leaf extract; LW = aqueous leaf extract; PC = turmeric positive control (1.25 mg/mL). Data is represented as mean values of triplicate results ± standard deviation. # and ## represent results that are significantly different to the negative control at *p* < 0.01 and *p* < 0.005, respectively

The production and secretion of cytokines is regulated by the transcription factor NF-κB (Serasanambati and Chilakapati 2016; Tripathi and Aggarwal 2006). Similarly, COX-2 synthesis (and, therefore, PGE_2_ secretion) are controlled by NF-κB (Serasanambati and Chilakapati 2016; Zarghi and Arfaei 2011). Therefore, the inhibition of cytokine, chemokine, COX-2 and PGE_2_ by the extracts may result from inhibition of NF-κB function. In contrast, LOX transcription is independent of NF-κB, although several LOX products are known to regulate NF-κB production, and thus affect the levels of other inflammatory mediators (Lorenzetti et al. 2019; Tavares et al. 2014). Therefore, the effects of the *T. ferdinandiana* extracts on cytosolic NF-κB levels in RAW 264.7 macrophages was also investigated. Interestingly, all the extracts significantly inhibited the cytosolic levels of NF-κB in the unstimulated RAW 264.7 macrophages (Fig. 6a). Similarly, the turmeric control also decreased cytosolic NF-κB significantly in the unstimulated cells. Notably, the methanolic extracts were substantially stronger inhibitors of NF-κB production than for the corresponding aqueous extracts.

The methanolic fruit and leaf extracts also significantly inhibited cytosolic NF-κB levels in LPS-stimulated RAW 264.7 macrophages, with decreases of approximately 33% and 44%, respectively, compared to the LPS-stimulated control (Fig. 6b). This compares favourably to the decrease in cytosolic NF-κB induced by the turmeric positive control (~ 34%). Curcumin (the main bioactive compound in turmeric) potently inhibits NF-κB transcription pathways (Ghasemi et al. 2019), and thereby down-regulates multiple pro-inflammatory mediator levels. It is, therefore, likely that the anti-inflammatory properties of the *T. ferdinandiana* extracts may also be mediated via suppression of NF-κB transcription. In contrast, the aqueous extracts did not significantly down-regulate the cytosolic levels of NF-κB in RAW 264.7 macrophages.

### Qualitative HPLC–MS/MS analysis of the methanolic *T. ferdinandiana* extracts

As the *T. ferdinandiana* methanolic fruit and leaf extracts displayed the greatest effects against the immune-modulatory and inflammatory mediators screened in this study, they were further examined by LC–MS analysis to highlight noteworthy components. Previous studies in our group developed analysis parameters to profile *T. ferdinandiana* extracts and identify noteworthy flavonoid and tannin components (Shalom and Cock 2018). The current study utilised a metabolomics fingerprint approach to detect these phytoconstituents, and to determine their relative abundances, which allows for a comparison between the constituents in the methanolic extracts. The presence of 12 previously highlighted compounds was verified in the methanolic fruit and leaf extracts (Table 2). The tannin components ellagic acid (and its dehydrate derivative), gallic acid, chebulic acid, corilagen and exifone were particularly abundant in both the fruit and leaf extracts. In addition, castalagin and chebulagic acid were abundant in the methanolic fruit extract, although they were detected in lower relative abundancies in the methanolic leaf extract, whilst punicalin and chebulinic acid were present in higher relative abundance in the leaf extract than in the fruit extract. The flavonoid rutin was abundant in both the fruit and leaf extracts.

**Table 2:** Qualitative HPLC–MS/MS analysis of the methanolic *T. ferdinandiana* fruit and leaf extracts in negative ionisation mode, elucidation of empirical formulas and identification of the compound

Putative identification	Empirical formula	Molecular mass	Retention time	Relative abundance (% total peak area)	
				Methanolic fruit extract	Methanolic leaf extract
Ellagic acid dihydrate	C_14_ H_10_ O_10_	338.0281	1.065	2.55	1.75
Gallic acid	C_4_ H_8_ O_5_	136.0374	1.383	4.7	4.3
Chebulic acid	C_14_ H_12_ O_11_	356.039	1.54	3.3	3.89
Ellagic acid	C_14_ H_6_ O_8_	285.1943	4.374	1.05	1.58
Castalagin	C_41_ H_26_ O_26_	934.0715	7.669	2.32	0.63
Corilagin	C_27_ H_22_ O_18_	634.082	7.774	2.83	2.43
Punicalin	C_34_ H_22_ O_22_	782.0621	8.501	0.54	1.79
Luteolin	C_21_ H_20_ O_11_	448.1025	8.604	0.46	0.23
Rutin	C_27_ H_30_ O_16_	610.521	8.73	1.26	2.84
Chebulinic acid	C_41_ H_32_ O_27_	956.113	8.872	0.87	1.87
Exifone	C_13_ H_10_ O_7_	278.0433	9.312	6.23	4.64
Chebulagic acid	C_41_ H_30_ O_27_	954.0965	9.878	1.13	0.44

Chromatograms were run in negative ionisation mode. % peak area refers to the peak area for the individual compound expressed as a % of the total area under peaks for the chromatogram in the relevant mode

## Discussion

*Terminalia ferdinandiana* fruit and leaves contain high levels of antioxidant molecules including tannins and flavonoids, many of which have been linked with the therapeutic properties of this species (Cock 2015). The antibacterial (Cheesman et al. 2019; Wright et al. 2019; McManus et al. 2017), antifungal (Noé et al. 2019) and anti-protozoal (Cock and Rayan 2020; Rayan et al. 2015) properties of *T. ferdinandiana* extracts have been relatively well reported. Similarly, several studies have reported anticancer properties for *T. ferdinandiana* fruit and leaf extracts, and have partially evaluated the therapeutic mechanism(s) (Shalom and Cock 2018; Tan et al. 2011a, b). In contrast, the inflammatory properties of *T. ferdinandiana* fruit and leaf extracts have been relatively poorly studied, whilst the immune-modulatory properties remain unreported. Indeed, only two studies that screened some *T. ferdinandiana* fruit extracts against some inflammatory biomarkers were found (Tan et al. 2011a, b). Those studies reported that fractionated *T. ferdinandiana* fruit extracts significantly decreased the levels of COX-2 in LPS-stimulated macrophages. The authors also reported that the fractions increased the cellular Nrf2/Keap1 ratio significantly and postulated that these effects were mediated via NF-κB modulation. Those studies only tested extracts and fractions prepared from the fruit, and the effects of leaf extracts/fractions/isolated compounds have been neglected to date. Our study extends the previous studies by screening *T. ferdinandiana* fruit and leaf extracts against pro- and anti-inflammatory cytokines and chemokines, and tests the effects on cytosolic COX-2 and NF-κB levels, and PGE_2_ and LTB_4_ secretion.

Herein, we report that *T. ferdinandiana* fruit and leaf extracts have potent immune-modulatory activity. Notably, the *T. ferdinandiana* fruit and leaf extracts were potent inhibitors of all the pro-inflammatory and anti-inflammatory cytokines tested. The methanolic extracts were particularly good modulators of cytokine and chemokine secretion, although the aqueous extracts also substantially reduced secretion from the RAW 264.7 cells. IFN-γ, IL-1β, IL-2, IL-6 and TFN-α were selected for this study as they are the major pro-inflammatory cytokines, whilst IL-2 may also have anti-inflammatory effects (Kany et al. 2019). IL-1β, IL-6 and TFN-α are particularly relevant, contributing to cancer and the majority of inflammatory disorders, including cardiovascular disorders, rheumatoid arthritis and several other autoimmune inflammatory diseases, pulmonary inflammation, as well as type-2 diabetes mellitus (Zhao et al. 2013; Turner et al. 2014; Jiang et al. 2020). IL-1β is secreted by monocytes and macrophages in response to microbial infection, cell injury and inflammation (Zhang and An 2007). It primarily exerts its affects via stimulation of CD4+ T cells (Dinarello and Van der Meer 2013). Notably, all the *T. ferdinandiana* extracts were potent inhibitors of IL-1β in our study. Indeed, both methanol extracts and the aqueous leaf extract completely inhibited IL-1β secretion from RAW 264.7 cells, whilst the aqueous fruit inhibited IL-1β secretion by ~ 96%.

IL-6 is an important mediator of fever and acute phase responses and stimulates numerous autoimmune and inflammatory diseases. It has also been implicated in cytokine cascades that occur in severe SARS-Cov2 infections (Zhao 2020). IL-6 is secreted by macrophages in response to specific microbial motifs called pathogen-associated molecular patterns (PAMPs), which bind to cell receptors (including Toll-like receptors), stimulating the Janus kinase-signal transducer and transcription (JAK-STAT), Ras/Raf kinases, and the mitogen activated protein kinase (MAPK) intracellular signalling cascades (as reviewed in Kaur et al. 2020). All the *T. ferdinandiana* fruit and leaf extracts tested herein substantially inhibited IL-6 secretion, although the methanolic extracts were stronger inhibitors of IL-6 secretion than the aqueous extracts.

Similar trends were noted for TNF-α secretion, with the methanolic fruit and leaf extracts almost completely blocking TNF-α secretion when tested at 2.5 ml/mL concentrations. The aqueous fruit and leaf extracts also inhibited TNF-α secretion, albeit to a substantially lower extent (~ 27–39% inhibition compared to the untreated control). In addition, the *T. ferdinandiana* fruit and leaf extracts significantly inhibited secretion of the other pro-inflammatory cytokines IL-2 and IFN-γ, indicating that the extracts may be particularly useful for reducing inflammation by decreasing secretion of a wide range of pro-inflammatory cytokines. Therefore, the extracts may inhibit the early-phase inflammatory events and decrease the effects of inflammation via reducing pro-inflammatory cytokine secretion. Interestingly, the *T. ferdinandiana* extracts also significantly down-regulated the secretion of the anti-inflammatory cytokine IL-10 from RAW 264.7 macrophages. This may reduce their ability to modulate inflammatory responses once they have been initiated, although further study is required to verify this.

Chemokines are also important mediators of inflammation as they induce leukocyte chemotaxis to the inflammation site. The production and secretion of several chemokines including MCP-1 and MIP-2a are up-regulated in multiple inflammatory conditions, including atherosclerosis, psoriasis and rheumatoid arthritis (Cranford et al. 2016). This study evaluated the effects of the *T. ferdinandiana* fruit and leaf extracts on the important chemokines MCP-1 and MIP-2a. All the extracts significantly inhibited the secretion of both chemokines, although the methanolic extracts were particularly potent. Indeed, the methanolic fruit and leaf extracts both completely inhibited the secretion of MCP-1 and MIP-2a, whilst the aqueous extracts inhibited secretion by 10–18% compared to the untreated control RAW 264.7 cells.

Cytokines facilitate the up- and/or down-regulation of multiple genes and transcription factors during inflammation, and therefore, they may have profound effects on other inflammatory mediators (Jiang et al. 2020). The roles of eicosanoids in inflammation and immune-modulation have been particularly well studied. Secreted cytokines stimulate arachidonic acid release from membrane lipids and induce its metabolism by the cyclooxygenase (COX) and lipoxygenase (LOX) enzyme systems to produce further inflammatory mediators including prostaglandins and leukotrienes (Calder 2020). Notably, prostaglandins can be synthesised by both the COX-1 and COX-2 systems, although inflammation only induces the COX-2 enzyme. In contrast, COX-1 is constitutively expressed. Therefore, inhibition of COX-2 (but not COX-1) expression is a target anti-inflammatory drug development (Hawkey 2001). The effects of the *T. ferdinandiana* extracts on COX-2 expression were, therefore, evaluated herein. Notably, all the fruit and leaf extracts significantly decreased cytosolic COX-2 levels in LPS-stimulated RAW 264.7 macrophages. The methanolic extracts were particularly good inhibitors of COX-2 expression, decreasing cytosolic COX-2 levels by 87% and 95% for the fruit and leaf methanolic extracts, respectively, compared to the untreated but LPS-stimulated control. The aqueous extracts also significantly inhibited COX-2 expression, although the inhibition was substantially less pronounced (47–53%).

COX-2 catalysis of arachidonic acid results in the production of prostaglandin endoperoxide H_2_, which may be further converted to a variety of prostaglandins, prostacyclins and thromboxanes, with the pathway dependant of specific signals and cell types. In macrophages, PGE_2_ is a major product of arachidonic acid metabolism (Calder 2020). PGE_2_ has potent pro-inflammatory effects by enhancing vascular permeability, thereby allowing neutrophils and macrophages to enter the site of an infection (Calder 2020; Samuchiwal and Boyce 2018). It also potentiates the pain response and regulates pro- and anti-inflammatory cytokine production (Miles et al. 2002). Therefore, suppression of PGE_2_ production and secretion is a target for anti-inflammatory therapy. Not surprisingly, the effects *T. ferdinandiana* fruit and leaf extracts on PGE_2_ secretion mirror the trends noted for COX-2 expression, with particularly potent inhibition of PGE_2_ secretion noted for the methanolic fruit and leaf extracts, with lower (although also potent) inhibition seen for the aqueous extracts.

Inhibition of the COX-2 enzyme may make higher levels of arachidonic acid available for other enzymatic systems, including the LOX system. LTB_4,_ a major product of LOX catalysis of arachidonic acid in macrophages, also has profound inflammatory effects. It enhances vascular permeability and attracts macrophages and neutrophils to the inflammatory site (Calder 2020). It also enhances secretion of IL-1β and IL-6, thereby increasing the inflammatory response (Brandt and Serezani 2017). Thus, inhibiting LTB_4_ secretion is also a target for anti-inflammatory drug development. The effects of the *T. ferdinandiana* fruit and leaf extracts on LTB_4_ secretion in RAW 264.7 cells did not follow the same trends as noted for PGE_2_ secretion. Notably, the methanolic *T. ferdinandiana* fruit and leaf significantly increased LTB_4_ in RAW 264.7 cells by approximately 87% and 58% compared to the untreated control for the fruit and leaf extracts, respectively. It is unclear whether these increases are due to specific enhancement mechanisms (e.g. up-regulation of LOX expression) or due to increased arachidonic acid flux through the LOX system, as the extracts down-regulate the COX-2 system. In contrast, the aqueous fruit and leaf extracts significantly reduced the secretion of LTB_4_, indicating anti-inflammatory effects. Notably, the aqueous *T. ferdinandiana* extracts were also substantially less potent COX-2 inhibitors, and this may contribute to the differential effects between the aqueous and methanolic extracts. The less potent inhibition of COX-2 by the aqueous extracts may have had only minor effects on the availability of arachidonic acid to the LOX system, although this remains to be verified.

The inhibition of a wide variety of inflammatory-associated proteins (pro-inflammatory and anti-inflammatory cytokines, chemokines, COX-2), and the subsequent secretion of pro-inflammatory lipids (PGE_2_, LTB_4_) indicates that the extracts may function by a down-regulation of transcriptase common for the expression of all the proteins tested. As NF-κB controls cytokine production (Serasanambati and Chilakapati 2016), as well as COX-2 expression (Ulivi et al. 2008), we also tested the effects of the *T. ferdinandiana* fruit and leaf extracts on cytosolic NF-κB levels. The fruit and leaf methanolic extracts significantly down-regulated cytosolic NF-κB levels by approximately 33% and 44%, respectively, compared to the untreated control. Treatment with the aqueous extracts also resulted in non-significant cytosolic NF-κB level decreases. These results indicate that the immune-modulatory and anti-inflammatory effects of the *T. ferdinandiana* extracts may be mediated via a generalised down-regulation of NF-κB-mediated transcription.

**Fig. 7:**
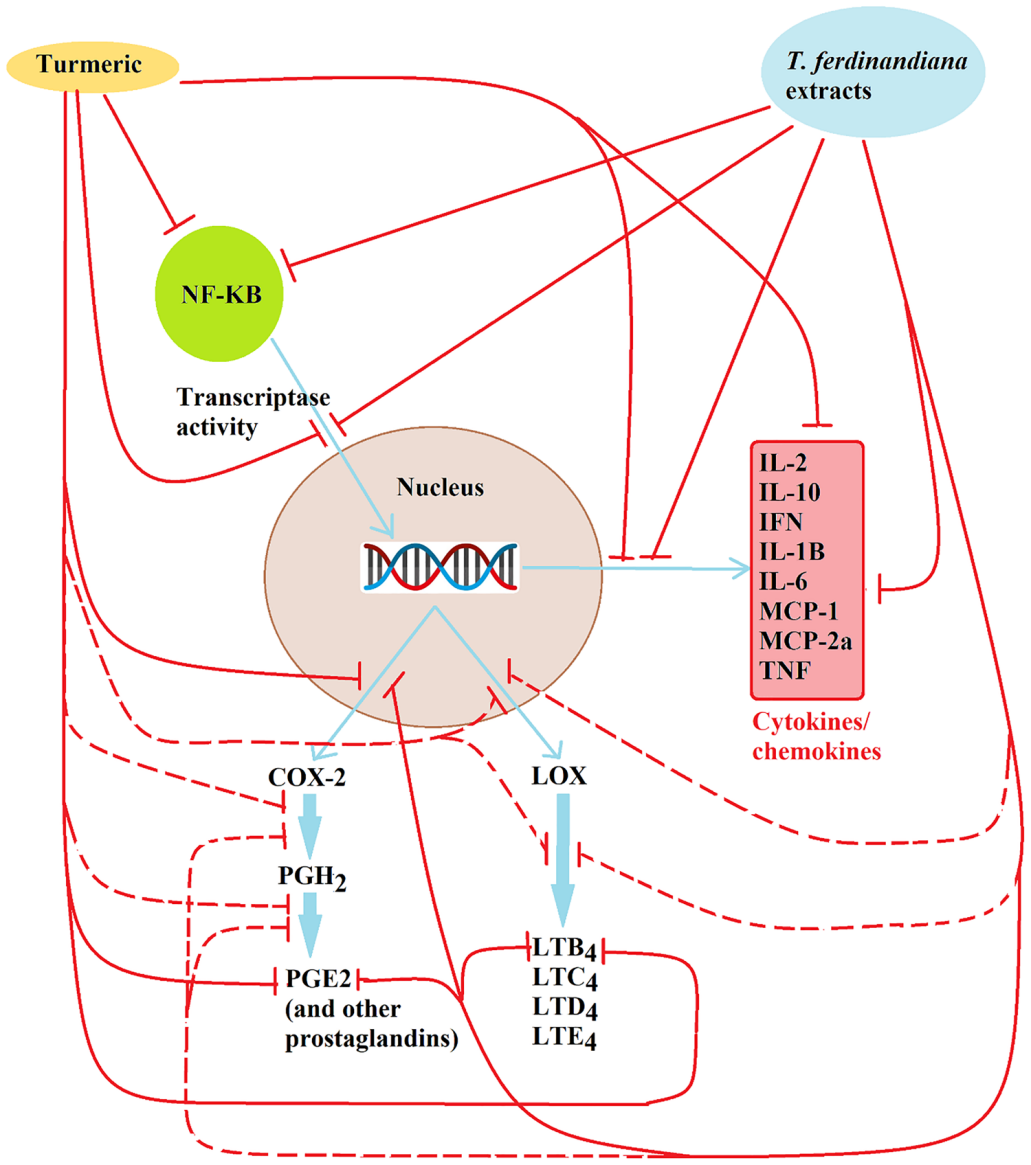
Proposed immune-modulatory and anti-inflammatory mechanisms of the *T. ferdinandiana* extracts and the turmeric control. Unbroken red lines indicate inhibitory activity demonstrated in this study; broken red lines indicate possible inhibitory activities not tested in this study, but consistent with the results. *T. ferdinandiana* extracts (and turmeric) down-regulate the levels of NF-κB in the cytosol. They also inhibit the production of the cytokines and chemokines, as well as COX-2, PGE_2_ and LTB_4_, indicating a general inhibition of transcription. The extracts (and turmeric control) may also directly inhibit the other mechanisms indicated, although this remains to be verified

From the results presented herein, a model for the immune-modulatory and anti-inflammatory activity of *T. ferdinandiana* extracts is proposed (Fig. 7). Components present in all the extracts directly down-regulate cytosolic NF-κB levels. The nature of this down-regulation remains to be determined, although tannin components of the extracts may be partially responsible for the decreased cytosolic NF-κB levels. Indeed, previous studies have confirmed that individual tannins (Karuppagounder et al. 2015) and tannin rich extracts (Ekambaram et al. 2022) have profound effects on cytosolic NF-κB levels. Regardless of the mechanism, the decreased levels of cytosolic NF-κB result in less NF-κB translocating into the nucleus, which subsequently down-regulates expression of the cytokines and chemokines, as well as COX-2. It is not known whether extract components also directly inhibit secretion of the cytokines, or COX-2 enzymatic activity. However, tannins have been reported to bind to proteins and inhibit the activity of several enzymes (Buzzini et al. 2008). Therefore, the *T. ferdinandiana* tannins may bind to COX-2 and LOX (and/or other enzymes in the COX and LOX pathways), thereby further reducing the production of PGE_2_ and LTB_4_, although this remains to be verified. Figure 7 also indicates other proposed mechanisms by which the extracts may down-regulate the secreted and cytosolic inflammatory mediators that are yet to be tested.

## Conclusions

*Terminalia ferdinandiana* has attracted considerable attention due to its promising antioxidant, antimicrobial and anticancer properties. However, the immune-modulatory and anti-inflammatory potential of *T. ferdinandiana* extracts have been largely neglected. *Terminalia ferdinandiana* fruit and leaf extracts were evaluated for their immune-modulatory properties via monitoring the effects on IL-2, IL-10, IFN-γ, IL-1β, IL-6, MCP-1, MIP-2a and TNF-α secretion in RAW 264.7 cells. This study reports for the first time that *T. ferdinandiana* fruit and leaf extracts potently inhibit secretion of multiple cytokines and chemokines. Furthermore, the results indicate that this immune-modulatory activity may be due to a reduction in cytosolic NF-κB levels. The extracts also down-regulated cytosolic COX-2 levels, subsequently decreasing PGE_2_ production. In addition, *T. ferdinandiana* fruit and leaf extracts also modulate LTB_4_ synthesis in RAW 264.7 macrophages, although the LTB_4_-modulatory mechanism(s) were not identified. Purification of the bioactive components and determination of the mechanisms is required to completely elucidate the immune-modulatory and anti-inflammatory properties of *T. ferdinandiana* fruit and leaves.

## Data Availability

Enquiries about data availability should be direscted to the author.
